# Preparation of PVDF-PVP Composite Membranes for Oily Wastewater Treatment

**DOI:** 10.3390/membranes13060611

**Published:** 2023-06-20

**Authors:** Sutrasno Kartohardjono, Ghofira Muna Khansa Salsabila, Azzahra Ramadhani, Irfan Purnawan, Woei Jye Lau

**Affiliations:** 1 Department of Chemical Engineering, Faculty of Engineering, Universitas Indonesia, Kampus UI, Depok 16424, Indonesia; ghofira.muna@ui.ac.id (G.M.K.S.); azzahra.ramadhani@ui.ac.id (A.R.); irfan.purnawan@ui.ac.id (I.P.); 2 Advanced Membrane Technology Research Center, Universiti Teknologi Malaysia, Johor 81310, Malaysia; lwoeijye@utm.my

**Keywords:** hydrophylicity, oily wastewater treatment, porosity, PVDF, PVP, UF

## Abstract

The oil and gas industry and related applications generate large quantities of oily wastewater, which can adversely affect the environment and human health if not properly handled. This study aims to prepare polyvinylidene fluoride (PVDF) membranes incorporated with polyvinylpyrrolidone (PVP) additives and utilize them to treat oily wastewater through the ultrafiltration (UF) process. Flat sheet membranes were prepared using PVDF dissolved in *N*,*N*-dimethylacetamide, followed by the addition of PVP ranging from 0.5 to 35 g. Characterization by scanning electron microscopy (SEM), water contact angle, Fourier transform infrared spectroscopy (FTIR), and mechanical strength tests were performed on the flat PVDF/PVP membranes to understand and compare the changes in the physical and chemical properties of the membranes. Prior to the UF process, oily wastewater was treated by a coagulation–flocculation process through a jar tester using polyaluminum chloride (PAC) as a coagulant. Based on the characterization of the membrane, the addition of PVP improves the physical and chemical properties of the membrane. The membrane’s pore size becomes larger, which can increase its permeability and flux. In general, the addition of PVP to the PVDF membrane can increase the porosity and decrease the water contact angle, thereby increasing the membrane’s hydrophilicity. With respect to filtration performance, the wastewater flux of the resultant membrane increases with increasing PVP content, but the rejections for TSS, turbidity, TDS, and COD are reduced.

## 1. Introduction

The oil and gas industry and related applications generate large quantities of oily wastewater, adversely affecting the environment and human health if not properly handled. Oily wastewater must be treated appropriately before being discharged into the environment. Various methods exist to treat oily wastewater, including gravity settling, hydrocyclones, air flotation, media filtration, and membrane separation [1]. Meanwhile, absorbent materials, such as porous polycarbonate monoliths and polyethylene/MXene aerogel, are considered excellent candidates for oil spills because of their highly efficient and pollution-free oil/water separation capacities [2,3]. Membrane filtration processes are often chosen for economic and environmental reasons for oily wastewater treatment. However, membranes have drawbacks regarding their short lifespan due to membrane fouling [4,5]. Nanofiltration and reverse osmosis membranes can remove ions, salinity, and macromolecules from water but are very susceptible to contamination. Therefore, a pretreatment process is typically conducted in ultrafiltration (UF), which can remove impurities that cause fouling on the nanofiltration membrane and reverse osmosis. Other combined processes that are also adopted to minimize this fouling problem in wastewater treatment are coagulation and membrane filtration [6,7].

Various types of membrane materials have been used in many separation processes. Polymer-based membranes are currently very often used for several membrane purposes, including microfiltration (MF), ultrafiltration (UF), nanofiltration (NF), and reverse osmosis (RO) [8]. Among the polymeric materials available, such as polysulfone (PSF), polypropylene (PP), polytetrafluoroethylene (PTFE), polyethylene (PE), and polyvinylidene fluoride (PVDF) [9,10], PVDF membranes are widely used to make UF and MF membranes for wastewater treatment because they have excellent mechanical, physical, and chemical stability, as well as excellent thermal stability [11]. Although PTFE has the same structure and advantages as PVDF, it has a higher density. In contrast, PVDF has a low density and higher porosity, generally indicating higher water flux [12]. In addition to being able to separate organic matter, including carbohydrates, proteins, and fats, PVDF membranes also have high oxidant tolerance, great mechanical strength, and excellent resistance to fouling. In addition, many common organic solvents can be used to dissolve it. These include *N*-dimethylacetamide (DMAc), tetraethyl phosphate (TEP), *N*-methyl-2-pyrrolidone (NMP), and dimethylformamide (DMF) [13,14]. Furthermore, PVDF has compatibility with other polymers, such as polyvinylpyrrolidone (PVP), in various mixed compositions, which is very useful in preparing membranes according to the desired criteria [15].

The immersion-phase precipitation technique is most widely applied to membrane preparations where the base, solvent, and non-solvent materials are primarily required. However, the combination of PVDF with DMAc produced a more potent solvent than other solvents. DMAc is a versatile solvent because of its high boiling point and good thermal and chemical stability, thus giving good membrane properties in terms of porosity, mechanical properties, and pure water flux [16]. Additives such as PVP can increase the surface hydrophilicity of the PVDF membrane. Based on several studies, PVP can increase the porosity of PVDF membranes and the amount of pure water flux. PVP is also a non-toxic substance with good solubility in water and solvents, making it a suitable polymer additive.

In view of this, this study aims to prepare and characterize PVDF membranes with PVP additives for the treatment of oily wastewater combined with the coagulation-flocculation process using poly aluminum chloride (PAC) as a coagulant. PAC is widely used for wastewater treatment [17]. Based on a previous study on the PVDF/PVP membrane for tofu industrial wastewater treatment [15], it is reported that upon addition of PVP, a membrane with good structure, excellent mechanical properties, and high porosity could be produced, in addition to increased surface hydrophilicity. Meanwhile, PAC has been successfully used in pretreatment for tofu and batik industrial wastewater treatment [18].

## 2. Materials and Methods

### 2.1. Membrane Preparation

All chemicals used for membrane preparation were analytical grade, and PVDF, PVP, and DMAC were purchased from Solvay Chemical USA, Sigma-Aldrich Indonesia, and Merck Indonesia, respectively. Deionized water and ethanol were obtained from Dwinika Intan Mandiri, Indonesia. The composition of the casting solution is shown in Table 1. The details of the flat sheet membrane preparation procedure can be found elsewhere [15].

### 2.2. Membrane Characterization

Several methods are applied to characterize changes in membranes’ physical and chemical properties, including scanning electron microscopy (SEM) analysis, Fourier transform infrared spectroscopy (FTIR), and membrane tensile tests. The surface morphology and cross-section of the membranes, the membrane surface’s chemical composition, and the membranes’ mechanical strength were characterized using SEM, ZEISS Ultra 60, Thermo Scientific FTIR, Diamond Nicolet IS 5, and Universal Testing Machine, UTM 10 kN, respectively. Meanwhile, the water contact angle and membrane porosity were characterized using a digital camera (Angle Meter) and the dry-wet mass method, respectively. Based on the dry-wet mass method, the membrane porosity, *e*, can be calculated by [19]:

(1) $$\varepsilon =\frac{m_{1}-m_{2}}{A\rho l}$$

where *m*_1_, *m*_2_, *A*, *ρ*, and *l* are the weights of wet membrane (g), dry membrane (g), membrane surface area (cm^2^), liquid density (g/cm^3^), and membrane thickness (cm), respectively.

### 2.3. Pretreatment of Oily Wastewater

Oily wastewater used as feed for the UF process must have a specific particle size because it can cause fouling and damage to the membrane. Therefore, it is necessary to do the pretreatment process through the coagulation–flocculation method in the jar test. This study used 500 ppm PAC for the coagulation–flocculation pretreatment [20]. The 500 ppm PAC dose was selected based on a previous study, which provided the optimum dose in the tofu industrial wastewater treatment process [5]. The coagulation–flocculation process was conducted using a jar test with fast stirring at 120 rpm for 2 min, followed by slow stirring at 40 rpm for 10 min. Furthermore, the samples were allowed to stand for 30 min before the oily water emulsion waste was filtered using filter paper and used as feed for the UF process. The main characteristics of the oily wastewater are presented in Table 1.

### 2.4. Ultrafiltration Test

Ultrafiltration tests for oily wastewater were conducted using the Amicon^®^ Stirred Cell UFSC05001 at 3 bar. The parameters measured for the resulting UF water are total suspended solids (TSS), total dissolved solids (TDS), turbidity, chemical oxygen demand (COD), and pH. Turbidity and TSS were measured using a Colorimeter DR/890, and pH and TDS were measured using a Hanna Combo pH meter. UF water flux and removal of oily wastewater parameters are calculated using Equations (2) and (3), respectively [15]:

(2) $$J=\frac{Q_{l}}{A_{m}}$$

(3) $$R=\frac{C_{in}-C_{out}}{C_{in}}$$

where *J*, *Q_l_*, *A_m_*, and *P* are water flux (L/m^2^.h), water permeation rate (L/h), and membrane area (m^2^), respectively. Meanwhile, *R*, *C_in_*, and *C_out_* are rejection, inlet concentration (mg/L), and outlet concentration (mg/L), respectively.

## 3. Results and Discussion

### 3.1. Membrane Characterization

**Figure 1:**
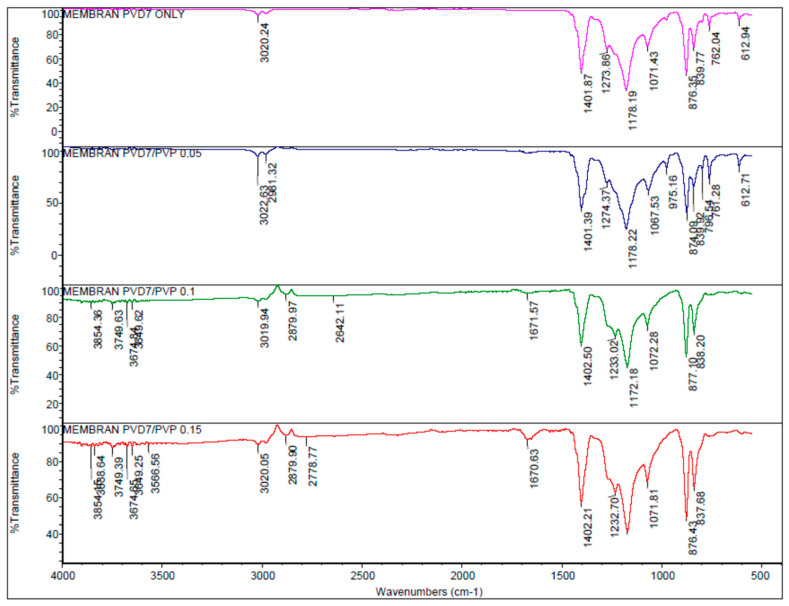
FTIR spectra of the prepared membranes.

Figure 1 presents the FTIR results for pristine PVDF and PVDF/PVP membranes with various PVP addition compositions. As shown, there is no peak in the PVDF (pristine) at a wavelength of 1670 cm^−1^. The peaks detected at wavenumbers of 876 cm^−1^ and 1270 cm^−1^ indicate the CF_2_ strain and CH_2_ bonds in the PVDF membrane. Very shallow valleys can be seen in other PVDF curves with various PVP additions at wavenumbers between 1631 and 1680 cm^−1^, proving that PVP exists in the composite membranes. This phenomenon indicates the stretching of the carbonyl bonds in PVP. The peak at PVDF/PVP 0.05 (M-05) was minimal because the concentration of PVP added was relatively low, namely PVDF/PVP 14.95/0.05 (0.33 by weight of PVDF). Generally, the functional groups indicating the presence of PVDF content in the membrane are known from the CH_2_ symmetrical and asymmetric stretching vibrations at wavenumbers around 2960 cm^−1^ and 3022 cm^−1^ [21]. CH_2_ deformation stretching vibrations are present at wavenumbers 1402 cm^−1^ and CF_2_ stretching and bending vibrations at wavenumbers 1174.5 cm^−1^ and 1067 cm^−1^ [22]. Meanwhile, the functional groups indicating the presence of PVP content in the membrane are known from the wavenumbers around 1665 cm^−1^ and 560 cm^−1^. These two peaks correspond to C=O and N-C=O in PVP, respectively. The wavenumber around 3620 cm^−1^ indicates the presence of O-H, which suggests there is ethanol bound to the PVP [23].

**Figure 2:**
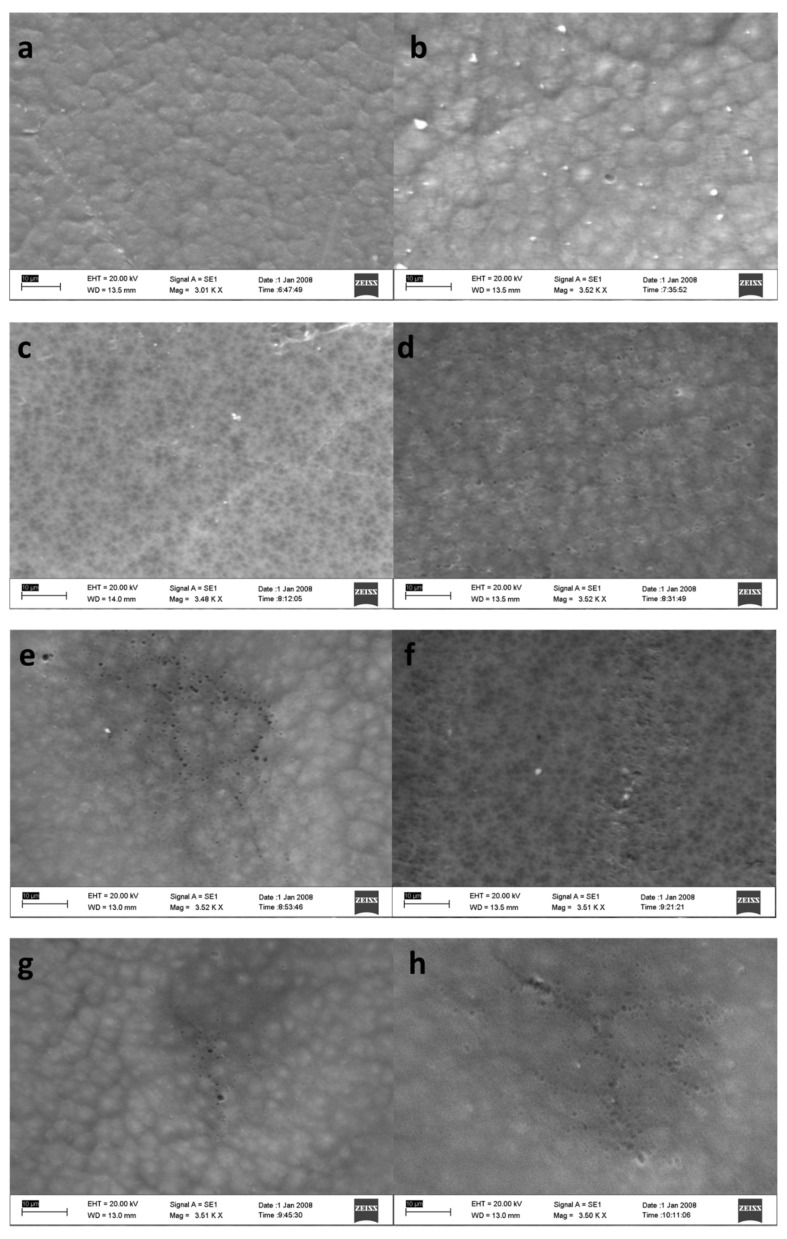
SEM images for pristine (**a**) M-05, (**b**) M-10, (**c**) M-15, (**d**) M-20, (**e**) M-25, (**f**) M-30, and (**g**) M-35 (**h**) membranes.

**Figure 3:**
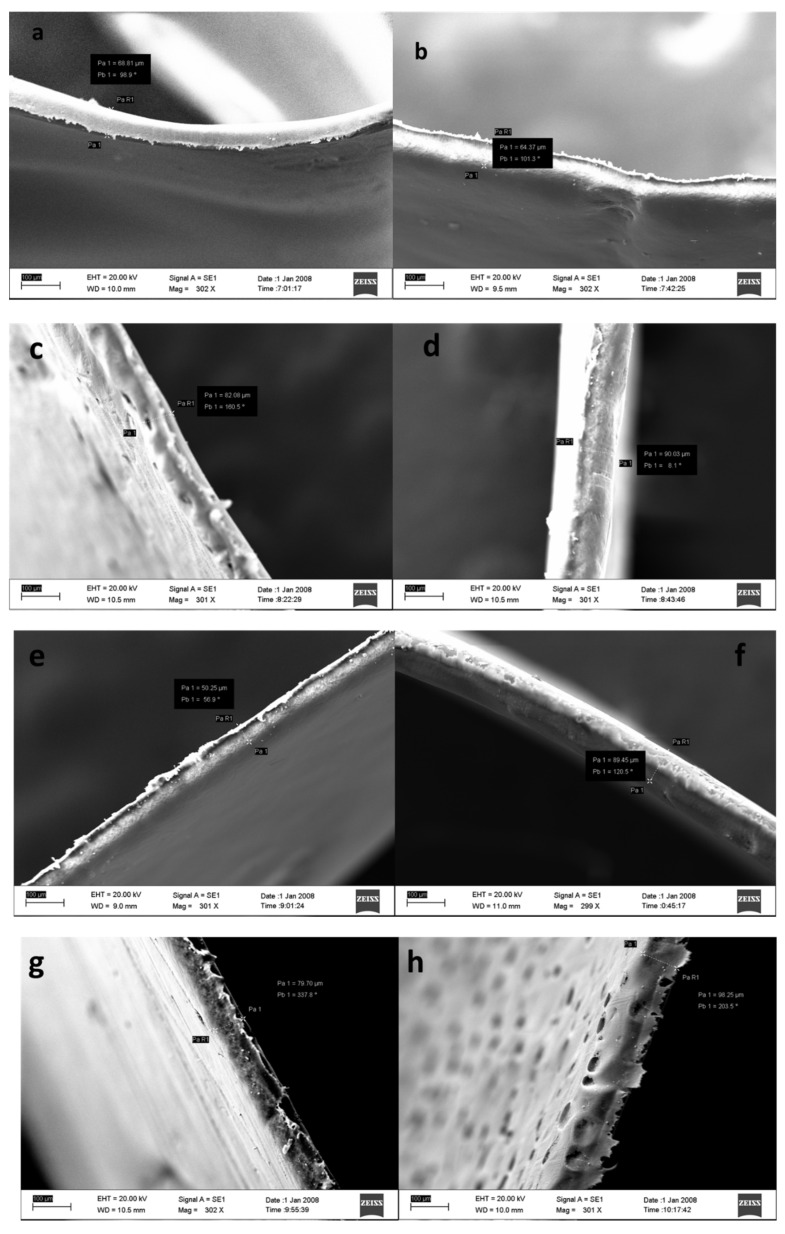
Cross-section images for pristine (**a**) M-05, (**b**) M-10, (**c**) M-15, (**d**) M-20, (**e**) M-25, (**f**) M-30, (**g**) M-35 and (**h**) membranes.

SEM results for the membrane surface are presented in Figure 2, which shows the distribution of pores on the surface of the membrane in each composition, and Figure 3 for the cross-section image. The membrane pores formed are indicated by white spots that spread over almost the entire membrane surface and are not the same size, indicating that the membrane is asymmetric. It can be seen from Figure 2 that the pore diameter in the PVDF/PVP0.05 (M-05) membrane is the smallest, with a small pore distribution. In contrast, the largest was obtained with an extensive distribution of the pore diameter in the PVDF/PVP0.35 (M-35) membrane. Membranes with a higher amount of PVP tend to have larger pore sizes than membranes prepared using a smaller amount of PVP. It is due to the role of PVP as a pore former in the phase inversion process of membrane formation. Adding more PVP could develop more pores with a larger diameter. Another cause of the PVDF/PVP0.35 (M-35) membrane having a larger pore size is the decrease in the amount of PVDF polymer between the PVDF/PVP0.35 (M-35) membrane and other membranes. The reduced polymer content could offer more space for an exchange of solvents and non-solvents, forming more pores.

Two-phase inversion mechanisms indicate the effects of adding additives on membrane structure and performance: the thermodynamic effect and the kinetic effect. With its thermodynamic effect, PVP can accelerate the change of solvent to non-solvent during the membrane coagulation process and increase the porosity of the membrane. As shown in Figure 3, the membrane thickness ranges from 50 to 97 μm. However, the average pore size is smaller. The average pore size of pure PVDF membranes cannot be identified because the membrane is dense, so the permeate value per unit of time cannot be determined. The kinetic effect increases the viscosity of the solution and causes the diffusion of solvents and non-solvents to occur more slowly. In this study, the thermodynamic impact of PVP is generally more dominant than the kinetic effect, resulting in membranes with a higher amount of PVP having larger pores and more pores in total.

**Figure 4:**
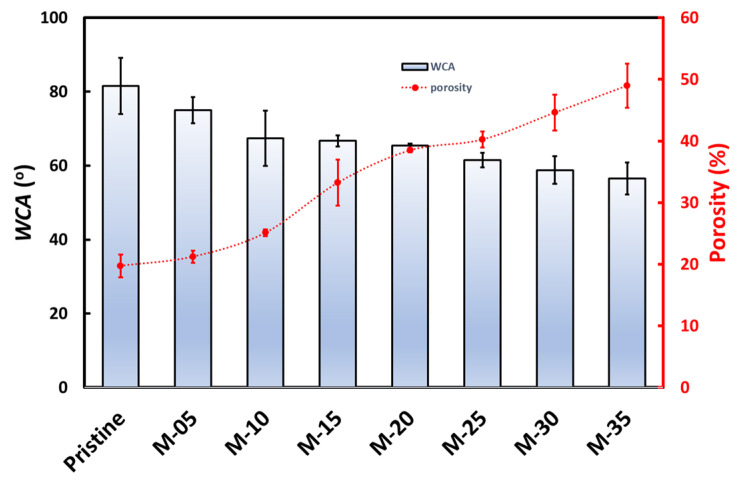
The water contact angle and the porosity of different types of PVDF membranes.

Figure 4 shows the porosity of the prepared membranes. Increasing the PVP can increase the porosity of the membrane because PVP is a pore-forming agent. In addition, adding PVP avoids a dense membrane surface and increases interpore penetration, which helps increase porosity. Figure 4 also shows that the pristine PVDF membrane has the lowest porosity (~20%), but the value is reported to increase as the PVP content rises. For the M-35 membrane, the porosity is found at ~49%.

The contact angle characterization results, as presented in Figure 4, show that the pristine membrane has a water contact angle of 81.5°, while M-05, M-10, M-15, M-20, M-25, M-30, and M-35 have water contact angles of 75°, 66.7°, 67.4°, 65.4°, 61.5°, 56.5°, and 58.8°, respectively. All the prepared membranes had a water contact angle below 90°, indicating that they all have hydrophilic properties. The higher the PVP composition in the membrane, the lower the contact angle, which suggests that adding more PVP resulted in a more hydrophilic membrane surface. Similar results have been reported previously, where the addition of PVP nanoparticles to the PVDF membrane can increase the hydrophilicity of the PVDF membrane due to the superhydrophilic PVP molecules, which affect the interaction between water molecules and the membrane surface [15]. Differences in membrane hydrophilicity can also be caused by differences in surface morphology, which can depend on the phase inversion process.

**Figure 5:**
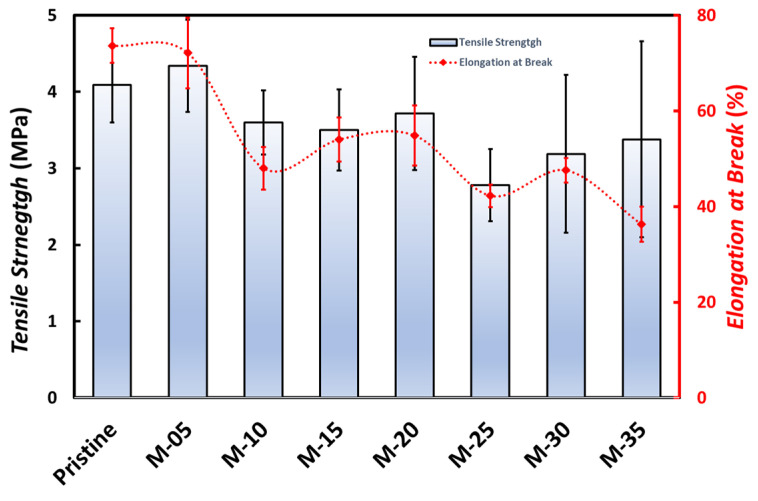
The tensile strength and the elongation at break of the PVDF/PVP membranes.

The tensile strength of the membrane, as shown in Figure 5, generally decreases with increasing PVP composition due to the expanded membrane pores formed. With increasing PVP, the elastic deformation of the membrane decreases, so that the membrane becomes more susceptible to swelling [24]. The tendency of the elongation percentage at the break is not the same as the tensile strength of the membrane. The decrease in elongation at the break on M-05 and M-35 and the increase in elongation at the break on M-15, as presented in Figure 5, show that the elongation percentage at the break can be affected by the viscosity of the doping solution [25].

### 3.2. Oily Wastewater Treatment

**Figure 6:**
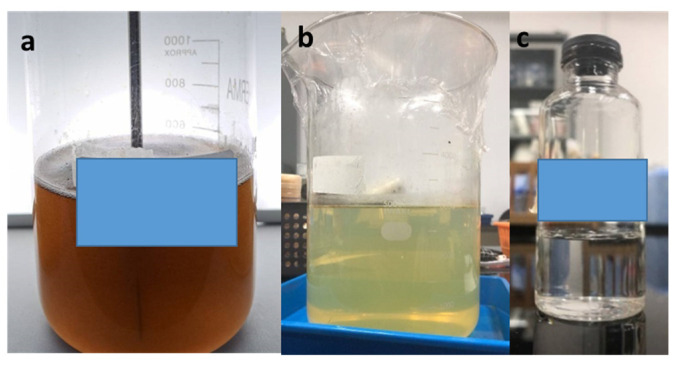
Oily wastewater emulsion (**a**) before and (**b**) after pretreatment using PAC and (**c**) after membrane process.

Figure 6 compares the oily wastewater before and after PAC pretreatment and the membrane process. The difference in turbidity can be very significant before and after the pretreatment and membrane processes. After pretreatment and the membrane process, the color of the oily wastewater was more transparent. A comparison of the characteristics of oily wastewater before and after the pretreatment process is shown in Table 2. The coagulation–flocculation process using a jar test was able to remove COD, TSS, and TDS and reduce the turbidity in an oily wastewater emulsion. Tiny particles are first destabilized by eliminating the colloids, leading to more extensive and heavier flocs forming. A stirring begins at a low speed in the coagulation–flocculation process, which helps shorten the distance between the particles so that the attractive forces between the particles become larger and more dominant than the repulsive forces, resulting in more frequent contact and collisions between the particles. It will agglomerate small-sized coagulated solid particles into large floc particles. After large flocs are formed, the waste will be precipitated for 30 min, and it can be filtered using filter paper [15]. In this study, the coagulation–flocculation pretreatment process proved effective, as seen from the TSS and turbidity removal. TDS removal is not very significant because the solids still present in the waste cannot be filtered by filter paper. These small or medium-sized solids, commonly called colloidal transitions, have dissolved into the sample and cannot be filtered out. The same result occurred with the COD data, which had an insignificant rejection compared to before pretreatment.

**Figure 7:**
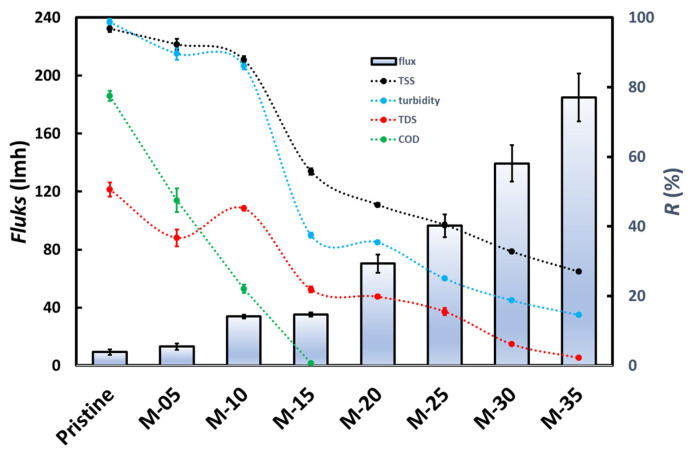
The oily wastewater flux and the rejection of TSS, turbidity, TDS, and COD of the PVDF-PVP membranes.

Figure 7 shows the flux of oily wastewater and the rejection of TSS, turbidity, TDS, and COD for various membranes at 3 bar. The oily wastewater flux increases with increasing PVP in the membrane. Adding PVP increases the membrane’s porosity and hydrophilicity, as shown in Figure 4, and ultimately improves the flux of oily wastewater through the membrane [26]. Figure 7 shows that the pristine PVDF membrane has the lowest flux of about 9 L/m^2^h and increases as the PVP content rises until around 185 L/m^2^h for the M-35 membrane.

TSS rejection, as presented in Figure 7, decreases with increasing PVP in the membrane, mainly due to the membrane’s pore size and hydrophobicity. In terms of pore size, such as the SEM characteristic that the membrane with the least PVP composition has the smallest pores, the suspended solids in the oily-water waste are more effectively retained in the pores and on the surface of the membrane. The second factor, hydrophobicity, also determines the percentage of TSS rejection. It can also be seen in the water contact angle test that a decrease in the PVP composition of the membrane leads to the membrane being more hydrophobic. This hydrophobic property makes the solids easier to filter because membranes tend to hold more water on the surface than membranes with less hydrophobicity, which can be seen in the oily-water waste flux section. Figure 7 shows that the pristine PVDF membrane has the highest TSS rejection of about 96.8% and decreases as the PVP content rises to around 26.9% for the M-35 membrane.

The turbidity rejection, as shown in Figure 7, is directly proportional to the TSS rejection. The turbidity parameter has a linear relationship with the TSS parameter because a high TSS value indicates many suspended particles in a solution, making the solution cloudy, especially if the suspended particles have a cloudier color than water. Another factor that can support high turbidity rejection is the tiny particles that pass through the membrane layer. These small particle sizes produce a more transparent effect on the permeate than large particle sizes, which can be more visible and increase the permeate’s turbidity. Figure 7 shows that the pristine PVDF membrane has the highest turbidity rejection of about 98.6% and decreases as the PVP content rises to around 14.6% for the M-35 membrane.

TDS rejection, as demonstrated in Figure 7, decreased with increasing PVP in the membrane material and was less compared to TSS or turbidity rejection due to much smaller particle sizes compared to suspended solid particle sizes. As shown in Figure 7, TDS rejection ranges from 2 to 48%, which is very low compared to TSS rejection. The solid particles in TDS are soluble, so they tend to have tiny particle sizes close to ionic sizes. The most effective removal of TDS can be achieved by reverse osmosis. TDS must be corrected in order for wastewater to be treated with it to meet quality standards because it is typically used to process drinking water. It can be seen from Figure 7 that the composition of the dope solution with less PVP composition tends to have a higher rejection because of its smaller pore size, although not small enough to reject TDS. In general, UF membranes are unsuitable for removing TDS because their pore size is much larger than the size of dissolved particles. These tiny particles are carried away during the UF process [27]. Figure 7 shows that the pristine PVDF membrane has the highest TDS rejection of about 50.5% and tends to decrease as the PVP content rises to around 2.5% for the M-35 membrane.

COD measures the amount of oxygen required so that organic and non-organic compounds in the wastewater can be oxidized by chemical reactions; high COD concentrations indicate the presence of high organic/non-organic contaminants [28]. COD rejection, as can be seen from Figure 7, decreased with increasing PVP in the membrane and was only significant for pristine, M-05, and M-10 membranes. Membranes with less PVP composition tend to provide better COD rejection due to their smaller pore sizes and larger contact angles to retain some organic compounds in oily wastewater. In this study, the synthetic oily water emulsion had a high value of around 99,136 mg/L. The high level of COD in synthetic oily wastewater is due to the Tween 80 emulsion mixture, which has many O-H groups, and these conditions are the same as those of industrial wastewater in general. Figure 7 shows that the pristine PVDF membrane has the highest turbidity rejection of about 77.5% and decreases as the PVP content rises to around 22.1% for the M-10 membrane. In contrast, the other membranes have no rejection for COD.

**Figure 8:**
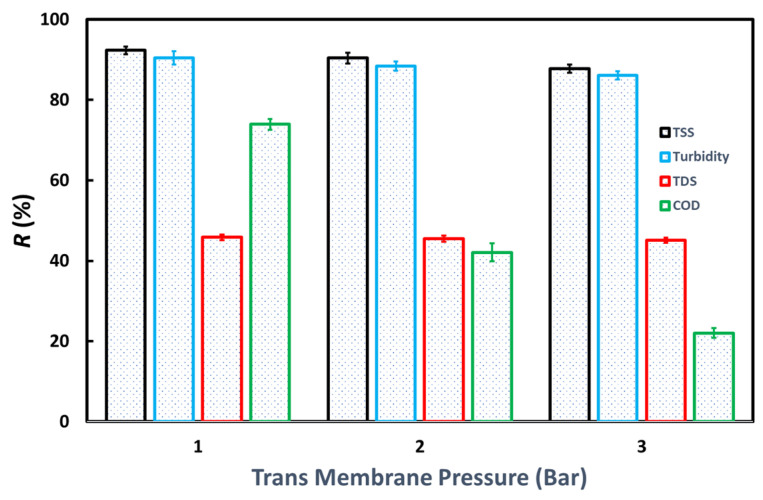
The effects of transmembrane pressure on the TSS, turbidity, TDS, and COD rejection of the M-10 membrane.

Based on Figure 7, the M-10 membrane is the best membrane because it can provide high rejection of TSS, turbidity, TDS, and COD and provides a flux that is not too low. Figure 8 shows the effect of transmembrane pressure (TMP) on the UF process for the M-10 membrane on TSS, turbidity, TDS, and COD removal. The rejection for the four parameters looks best at low TMP. It means that at a low TMP, the membrane performs better in retaining the four parameters tested. Meanwhile, more chemical substances can penetrate the membrane at a higher TMP due to increased driving force [5].

## 4. Conclusions

The experiments have been conducted to prepare the PVDF/PVP composite membranes through the phase inversion process and to characterize the membranes for oily wastewater treatment. The flat sheet membranes were prepared using PVDF dissolved in DMAc and then added with PVP additives, with variations in the composition of 0.5–35 g PVP. Based on the characterization of the membrane, the addition of PVP improves the physical and chemical properties of the membrane. The membrane’s pore size becomes larger, which can increase its permeability and flux. Generally, adding PVP to PVDF can increase the porosity and decrease the water contact angle, thereby increasing the membrane’s hydrophilicity. The tensile strength generally reduces with increasing PVP composition in the membrane due to the increased membrane porosity. The tendency of the percentage of elongation at break is not similar to the tensile strength of the membrane, which the viscosity of the doping solution can influence. Wastewater flux in the UF process increased with increasing PVP in the membrane material but decreased rejection for TSS, turbidity, TDS, and COD.

## Figures and Tables

**Table 1:** The main characteristics of oily wastewater.

Parameter	Value
TSS (mg/L)	194 ± 3.3
Turbidity (NTU)	185 + 3.5
TDS (mg/L)	10,280 ± 681
COD (mg/L)	99,216 ± 7071
pH	7.1 ± 0.2

**Table 2:** The oily wastewater characteristics before and after PAC pretreatment.

Parameter	Before Pretreatment	After Pretreatment
TSS (mg/L)	194 ± 3.3	52 ± 0.9
Turbidity (NTU)	185 + 3.5	48 ± 0.9
TDS (mg/L)	10,280 ± 681	9780 ± 648
COD (mg/L)	99,216 ± 7071	98,875 ± 5290
pH	7.1 ± 0.2	6.2 ± 0.2

## Data Availability

The data presented in this study are available upon request from the corresponding author. The data are not publicly available due to restrictions from the oil company.
